# Do NSAIDs affect bone healing rate, delay union, or cause non-union: an updated systematic review and meta-analysis

**DOI:** 10.3389/fendo.2024.1428240

**Published:** 2024-09-10

**Authors:** Po-Yao Chuang, Tien-Yu Yang, Yao-Hung Tsai, Kuo-Chin Huang

**Affiliations:** ^1^ Chang Gung University College of Medicine, Taoyuan, Taiwan; ^2^ Department of Orthopaedic Surgery, Chiayi Chang Gung Memorial Hospital, Chiayi, Taiwan

**Keywords:** nonsteroidal anti-inflammatory drugs (NSAIDs), cyclooxygenase-2 (COX-2) inhibitor, bone healing, non-union, delayed union

## Abstract

**Introduction:**

Nonsteroidal anti-inflammatory drugs (NSAIDs) may potentially delay or cause non-union of fractures by inhibiting prostaglandin synthesis. However, studies have shown conflicting results. This systematic review and meta-analysis aim to synthesize current evidence on the potential influence of NSAIDs on bone healing.

**Methods:**

We conducted a comprehensive search of PubMed, Embase, and Cochrane CENTRAL databases for studies published up to 25 July 2023. Specific keywords included “NSAID,” “nonsteroidal anti-inflammatory drug,” “cyclooxygenase-2 inhibitor,” “bone healing,” “non-union,” “pseudoarthrosis,” “delayed union,” and “atrophic bone.” Eligible studies included prospective, retrospective, and case-controlled studies assessing the correlation between NSAID use and bone healing outcomes. The leave-one-out approach was used to test the robustness of the meta-analysis results.

**Results:**

A total of 20 studies with 523,240 patients were included in the analysis. The mean patient age ranged from 6.7 to 77.0 years, with follow-up durations from 3 to 67 months. The meta-analysis revealed no significant difference in non-union or delayed union between NSAID users and non-users [pooled adjusted odds ratio (OR) = 1.11; 95% confidence interval (CI): 0.99–1.23]. Initial analysis identified a significant association between NSAID usage and an increased risk of reoperation, but this association became insignificant upon sensitivity analysis (crude OR = 1.42; 95% CI: 0.88–2.28).

**Discussion:**

NSAIDs may have a minimal impact on non-union or delayed union risks. However, caution is advised due to the limited number of studies and the absence of a specific focus on NSAID types and dosages. Further research is necessary to better understand the implications of NSAID use on bone healing.

## Introduction

Non-union is a condition where fractured bones fail to heal properly, and, in the United States, approximately 100,000 fractures annually result in non-union. The overall incidence of fracture non-union ranges from 1.9% to 10%, with variations depending on the bone involved. For instance, femoral shaft non-unions occur at an estimated rate of 8% when treated with intramedullary nailing. The tibial shaft non-union rate is reported to be 4.6% following the same treatment, although this figure is subject to variation as some studies suggest that tibia non-union rates could be as high as 10% to 12% (Thomas and Kehoe). Non-union of fractures is influenced by a variety of factors, including the nature and location of fracture, characteristics of patient, and the treatment methods. Fractures in specific bones, such as tibia, femur, and humerus, are especially susceptible to non-union, with open fractures being at a particularly high risk (1). Older age, smoking, poor nutrition, and medical conditions like diabetes and osteoporosis increase the risk, as do certain treatment-related factors like improper bone alignment and inadequate stabilization (Thomas and Kehoe; 2). Biological factors, including impaired blood supply to fracture site, can also play a role (3). Lifestyle choices and medications, such as alcohol abuse and corticosteroids, may further impair bone healing. Early detection and management, which may involve surgical interventions and lifestyle modifications, are key to treating non-union effectively (2).

Nonsteroidal anti-inflammatory drugs (NSAIDs) constitute a class of medications approved by Food and Drug Administration (FDA) for their antipyretic, anti-inflammatory, and analgesic properties (4). They effectively manage conditions like muscle pain, dysmenorrhea, arthritis, pyrexia, gout, and migraines and are used as opioid-sparing agents in acute trauma cases (5, 6). However, there has been ongoing debate about their potential impact on bone healing processes (7). Previous studies have suggested that NSAIDs might interfere with bone repair mechanisms by inhibiting prostaglandin synthesis, which could potentially lead to delayed union or non-union of fractures (8–11). However, there are contradictory results in the existing literature. Whereas some studies have identified detrimental effects on bone healing, others have not observed any significant association (11, 12). Variability in study designs, populations, and methodologies has contributed to these inconsistencies (13).

Given the ongoing discourse and the critical role of NSAIDs in pain management, an updated and comprehensive review is imperative to determine their potential effects on bone healing rates and outcomes. Clarifying the relation between NSAIDs and bone healing can help to provide informed decision-making in orthopedic and trauma care. Accordingly, this systematic review and meta-analysis aims to gather and synthesize current evidence on the potential influence of NSAIDs on bone healing.

## Methods

### Search strategy

The current systematic review and meta-analysis adhered to the Preferred Reporting Items for Systematic Reviews and Meta-Analyses (PRISMA) guidelines (14). PubMed, Embase, and Cochrane CENTRAL databases were search for relevant studies published up to 25 July 2023. The specific keywords used were “NSAID,” “nonsteroidal anti-inflammatory drug,” “cyclooxygenase-2 inhibitor,” “bone healing,” “non-union,” “nonunion,” “non union,” “pseudoarthrosis,” “delayed union,” “ununited,” and “atrophic bone.” Keywords were combined with Boolean operators and Medical Subject Headings (MeSH) terms where appropriate. An example search string used for PubMed was the following:

*(NSAID OR Nonsteroidal anti-inflammatory drug OR cyclooxygenase-2 inhibitor OR Cox-2 inhibitor) AND (bone healing OR non-union OR nonunion OR non union OR pseudoarthrosis OR delayed union OR ununited OR atrophic bone)*.

In addition, we conducted a manual search of the reference lists in the included studies to identify any potentially relevant research.

### Study selection criteria

This systematic review and meta-analysis was conducted following the PECOS criteria, which encompass participants (P), exposure (E), comparisons (C), outcomes (O), and study design (S). The inclusion criteria were as follows: studies participants with fractures, osteotomy, or those who underwent an orthopedic surgery (P); compared patients who were exposed to NSAIDs to those without exposure to NSAIDs (E&C). Outcomes of interest were bone healing rate, delayed union or non-union rate, or healing time (O). The eligible study designs included prospective and retrospective cohort studies, as well as case-controlled studies (S).

We excluded review articles, letters, commentaries, editorials, proceeding research, meeting abstracts, case reports, personal communications, non-English, and non-human studies. Eligibility of each study was confirmed by two independent reviewers, with a third reviewer consulted for uncertain cases.

### Main outcome measures and data extraction

The outcomes of interest centered on the association between NSAID usage and non-union/delayed union and the association between NSAID usage and reoperation (i.e., revision surgery).

From the eligible studies, we extracted the following information: the first author’s name, publication year, study design, total number of patients, type of NSAIDs, age, male (%), patient condition, and follow-up duration.

### Ethics statement

The systematic review and meta-analysis conducted in this study did not utilize raw patient data or private information. As a result, there was no requirement for further approval or informed consent from study subjects by the Institutional Review Board.

### Quality assessment

The quality of the included studies was assessed using the Newcastle–Ottawa scale (NOS), following the recommendations of the Cochrane Non-Randomized Studies Methods Working Group (15). The NOS assigns a maximum of nine points to each study: four points for appropriate selection of cohort participants, two points for comparability of participants in terms of design and analysis, and three points for adequate outcome ascertainment. Two independent reviewers conducted the quality assessment, and a third reviewer was consulted to resolve any uncertainties.

### Statistical analysis

In this research, the primary outcomes were non-union or delayed union and reoperation. Both crude odds ratios (ORs) and adjusted ORs (aORs) were used as the pooled effect estimates. Heterogeneity among the studies was evaluated using the Cochran Q test and I^2^ statistic. Heterogeneity was defined as follows: I^2^ ≤ 25%, low heterogeneity; 25% < I^2^ < 50%, moderate heterogeneity; 50% < I^2^ < 75%, substantial heterogeneity; and I^2^ ≥ 75%, high heterogeneity. The pooled estimates, 95% confidence interval (CI), and P-value were calculated using the random-effects model. All analyses were two-sided, with a significance level of α = 0.05. Publication bias was evaluated by using Funnel plots with Egger’s test. A sensitivity analysis was conducted using the leave-one-out approach. Subgroup analyses by age group (adults or children) and patient conditions were performed. All analyses were conducted using R Studio 4.3.2 with the packages “meta,” “dmetar,” and “metafor.”

## Results

### Study selection

**Figure 1:**
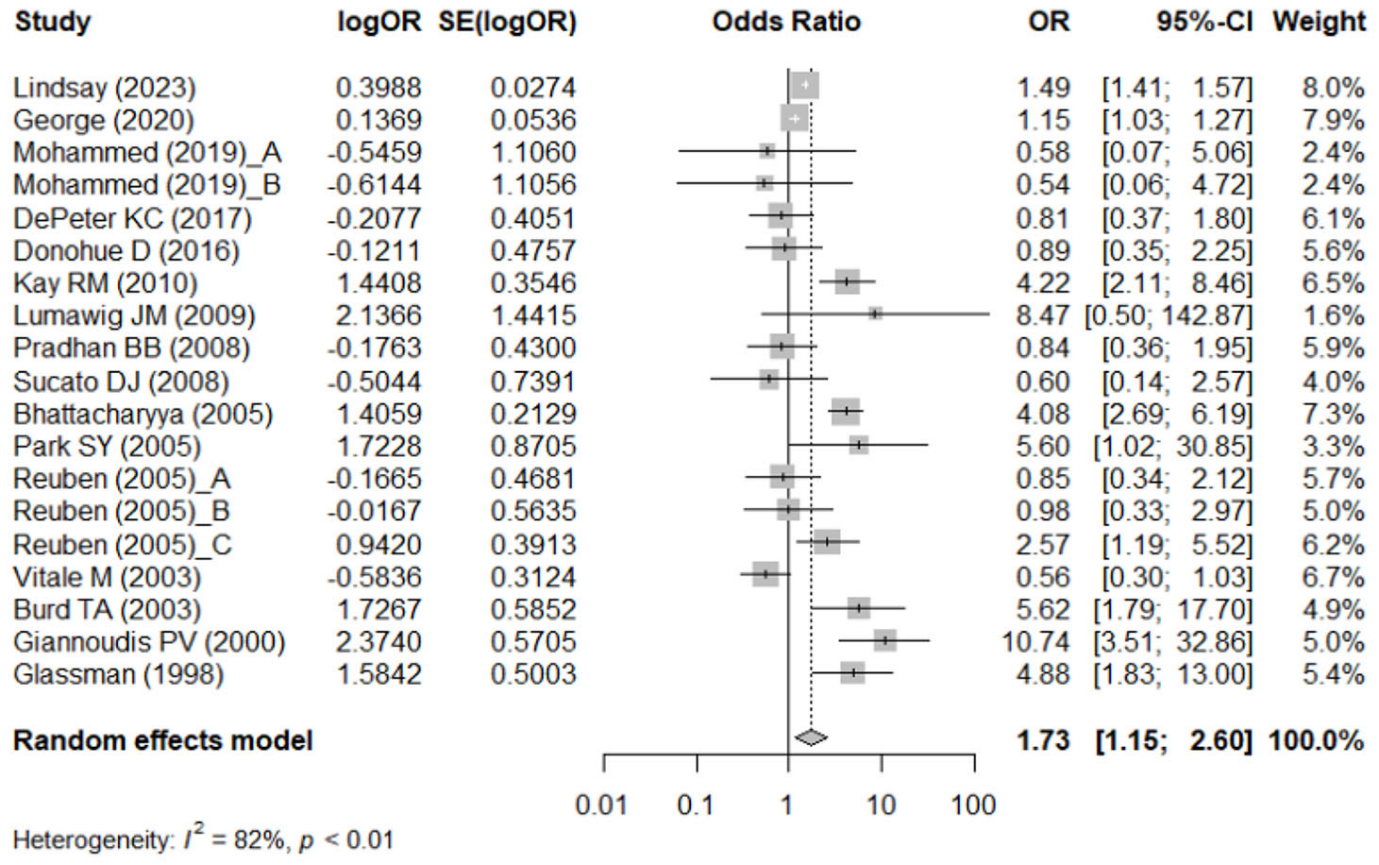
PRISMA flow diagram of study selection. The numbers of search hits corresponding to each step of the systematic literature search, qualitative review, and quantitative analysis are shown. The reasons for exclusion are described.

A flow diagram of the study selection process is shown in **Figure 1**. A total of 37 full-text articles were assessed for eligibility, and 17 were excluded for no quantitative outcome of interest (n = 10), duplicate patient population (n = 2), and study objectives not consistent with the aims of our analysis (n = 5). Consequently, 20 studies (16–35) containing a total of 523,240 patients were included in the qualitative and quantitative analysis (**Figure 1**).

### Characteristics of included studies

The study characteristics are summarized in **Table 1**. All studies were of retrospective design. The mean patient age ranged from 6.7 to 77.0 years, and the follow-up duration ranged from 3 to 67 months (**Table 1**).

**Table 1:** Characteristics of the included studies.

Study	Total number of patients	Number of patients and type of NSAID	Age, mean	Male, %	Patient condition	Follow-up duration, months	NOS
Lindsay (2023) (35)	178,758	Non-user: 155,156 Miscellaneous NSAIDs: 23,602	61.7	43.3	Degenerative spine disease	50	6/9
George (2020) (33)	326,876	Non-user: 279,720 Miscellaneous NSAIDs: 22,590	54.8	34.9	Long bone fracture	12	6/9
Mohammed (2019) (34)	232	Non-user: 173 Ibuprofen: 59 Non-user: 170 Ketorolac: 62	53.7	60.8	Foot and ankle disease	3	7/9
DePeter (2017) (32)	808	Non-user: 470 Ibuprofen: 338	9.49	63.3	Extremity fractures	NA	7/9
Donohue (2016) (31)	328	Non-user: 243 Ketorolac: 85	42.2	63.7	Femoral and tibial fractures	12	7/9
Blomquist (2014) (29)	477	Non-user: 322 Miscellaneous NSAIDs: 155	28.8	67.3	Shoulder instability	12	7/9
Jeffcoach (2014) (30)	1,901	Non-user: 1,670 Miscellaneous NSAIDs: 231	46.6	56.0	Long bone fracture	24	6/9
Schemitsch (2012) (28)	1,226	NA	39.5	NA	Long-bone fracture (tibia)	12	7/9
Kay (2010) (27)	221	Non-user: 52 Ketorolac: 169	6.7	48.0	Unspecified fractures	6	7/9
Lumawig (2009) (26)	273	Non-user: 19 Diclofenac: 254	60.0	47.0	Local autogenous bone graft	24	7/9
Pradhan (2008) (24)	405	Non-user: 177 Toradol: 228	56.2	35.8	Degenerative spine disease	30	7/9
Sucato (2008) (25)	319	Non-user: 161 Ketorolac: 158	14.3	15.5	Adolescent idiopathic scoliosis	39	6/9
Bhattacharyya (2005) (21)	9,995	Non-user: 8,963 Miscellaneous NSAIDs: 1,032	77.0	NA	Long bone fracture	12	6/9
Park (2005) (22)	88	Non-user: 58 Ketorolac: 30	52.7	32.6	Lumbar spinal degenerative disease	24	7/9
Reuben (2005) (23)	434	Non-user: 130 Rofecoxib: 124 Celecoxib: 60 Ketorolac: 120	46.1	58.5	Degenerative spine disease	12	7/9
Vitale (2003) (20)	208	Non-user: 148 Ketorolac: 60	13.4	28.9	Scoliosis	67	7/9
Bhandari M (2003) (18)	192	Non-user: 148 Miscellaneous NSAIDs: 44	38.0	73.0	Tibial shaft fracture	12	7/9
Burd (2003) (19)	112	Non-user: 74 Indomethacin: 38	38.6	NA	Long bone fracture	NA	6/9
Giannoudis (2000) (17)	99	Non-user: 70 Miscellaneous NSAIDs: 29	38.0	NA	Femoral diaphysis	NA	7/9
Glassman (1998) (16)	288	Non-user: 121 Ketorolac: 167	43.8	61.2	Degenerative spine disease	24	7/9

NSAIDs, non-steroidal anti-inflammatory drugs; NOS, Newcastle–Ottawa scale; NA, Not Applicable.

### Meta-analysis

#### Association between NSAID usage and non-union/delayed union assessed by unadjusted OR

**Figure 2:**
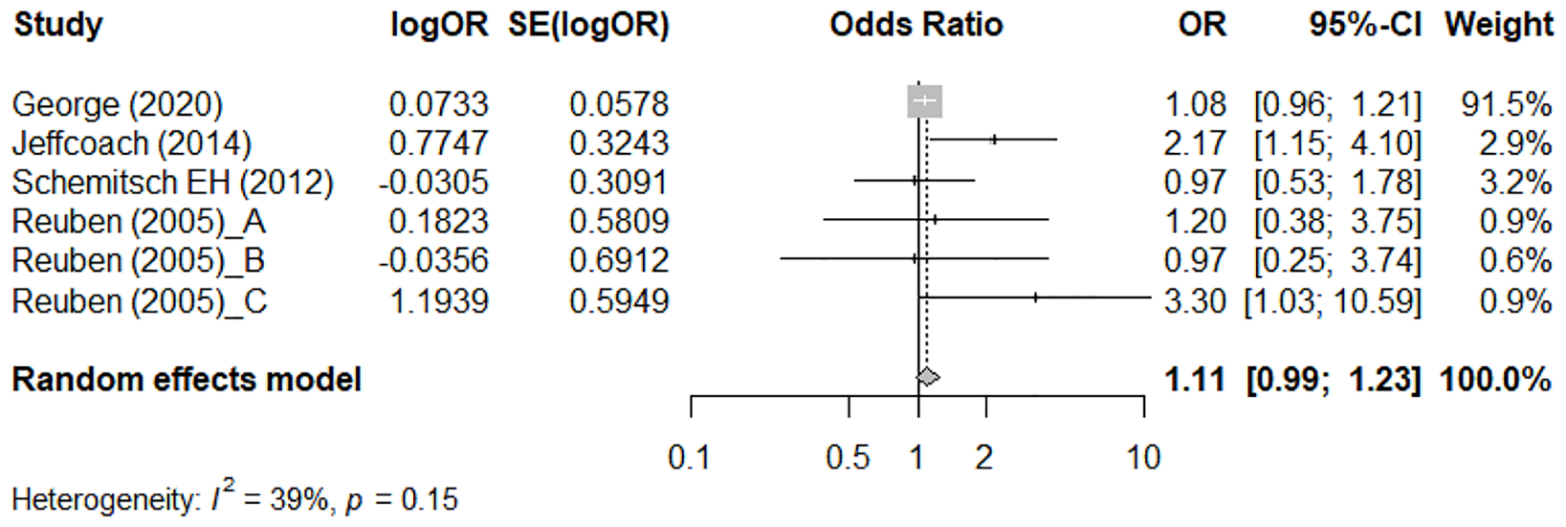
Meta-analysis of the association between NSAIDs usage and non-union/delayed union, assessed by crude odds ratio (OR).

**Figure 2** shows the meta-analysis results using the crude OR to examine the association between NSAID usage and non-union/delayed union. There were 16 studies (16, 17, 19–27, 31–35) that provided the crude OR or the necessary data to compute the crude OR. High heterogeneity across the studies was detected (I^2^ = 82%). Compared with non-users, NSAID users had a significantly higher risk of non-union/delayed union (pooled crude OR = 1.73; 95% CI: 1.15–2.60) (**Figure 2**).

Among adults, patients who were using NSAIDs had a significantly higher risk of non-union/delayed union (pooled crude OR = 1.98; 95% CI: 1.29–3.06; I^2^ = 82%). However, no significant association between NSAID usage and the risk of non-union/delayed was observed in children (pooled crude OR = 1.09; 95% CI: 0.46–2.59; I^2^ = 85%) (**Supplementary Figure 1**).

Among the subgroup of patients with a long bone fracture, those who were using NSAIDs had a significantly higher risk of non-union/delayed union (pooled crude OR = 2.27; 95% CI: 1.07–4.82; I^2^ = 91%). For patients receiving treatment for a degenerative spine fracture, NSAID usage was significantly associated with increased risk of non-union/delayed union (pooled crude OR = 1.49; 95% CI: 1.41–1.57; I^2^ = 64%). Among patients with scoliosis, those who were using NSAIDs had a lowered risk of non-union/delayed union in scoliosis (pooled crude OR = 0.56; 95% CI: 0.32–0.99; I^2^ = 0%) (**Supplementary Figure 2**).

#### Association between NSAID usage and non-union/delayed union assessed by adjusted OR

**Figure 3:**
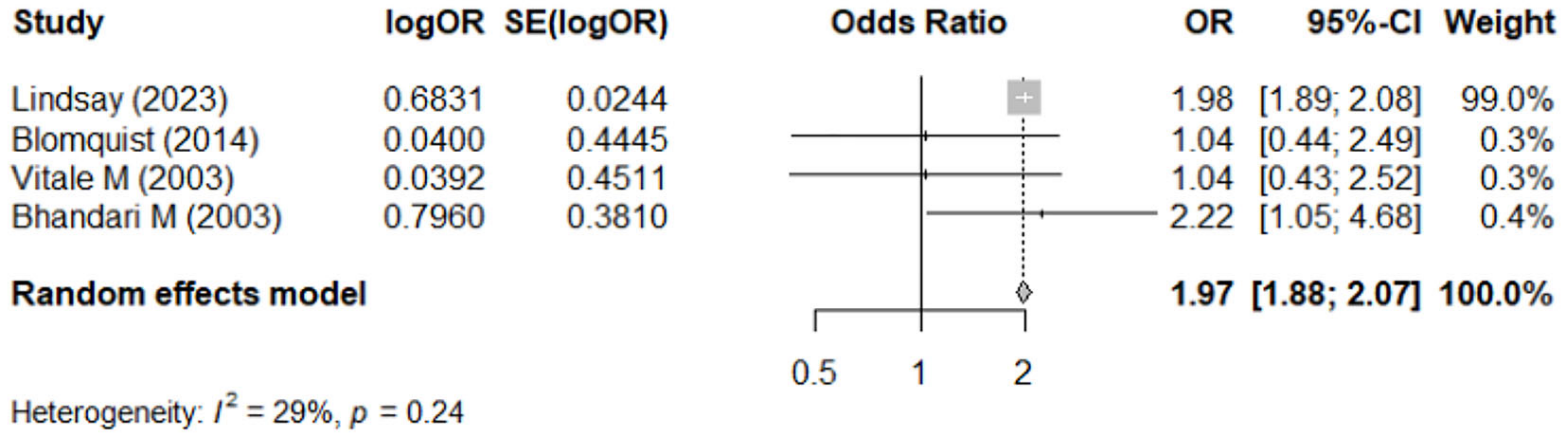
Meta-analysis of the association between NSAIDs usage and non-union/delayed union, assessed by adjusted odds ratio (OR).

**Figure 3** shows the meta-analysis results using the adjusted OR to assess the association between NSAID usage and non-union/delayed union. There were four studies (23, 28, 30, 33) that provided an adjusted OR. Moderate heterogeneity across the studies was detected (I^2^ = 39%). There was no significant difference in the rates of non-union or delayed union between NSAID users and non-users (pooled adjusted OR = 1.11; 95% CI: 0.99–1.23) (**Figure 3**).

#### Association between NSAID usage and reoperation assessed by unadjusted OR

**Figure 4:**
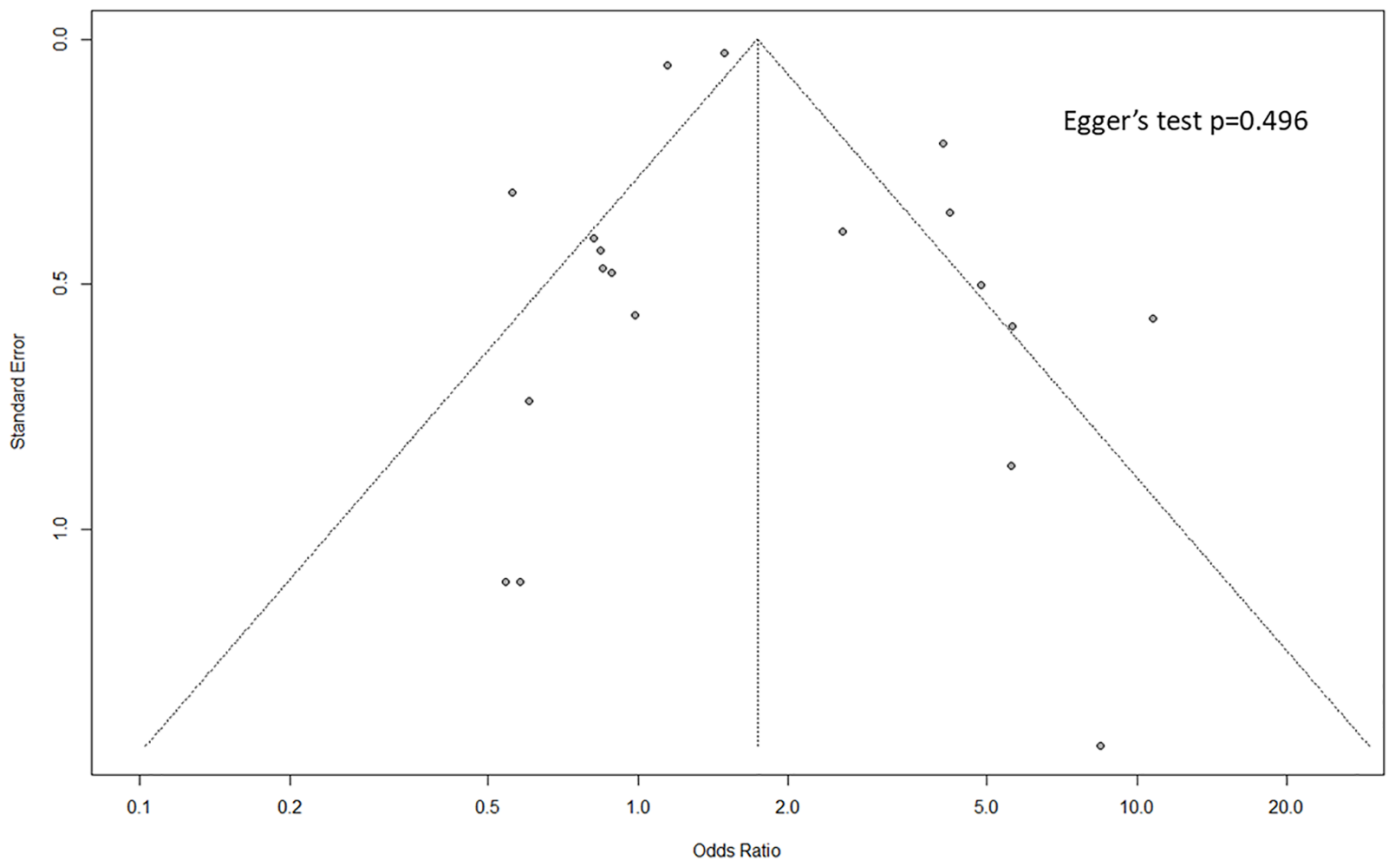
Meta-analysis of the association between NSAIDs usage and reoperation, assessed by crude odds ratio (OR).

**Figure 4** demonstrates the result of the meta-analysis on the association between NSAID usage and risk of reoperation, represented by crude OR. Only four studies (18, 20, 29, 35) reported relevant data, and moderate heterogeneity was detected among the studies (I^2^ = 29%). Compared with non-user, NSAID users had a significantly higher risk for reoperation (pooled crude OR = 1.97; 95% CI: 1.88–2.07) (**Figure 4**).

### Publication bias

**Figure 5:**
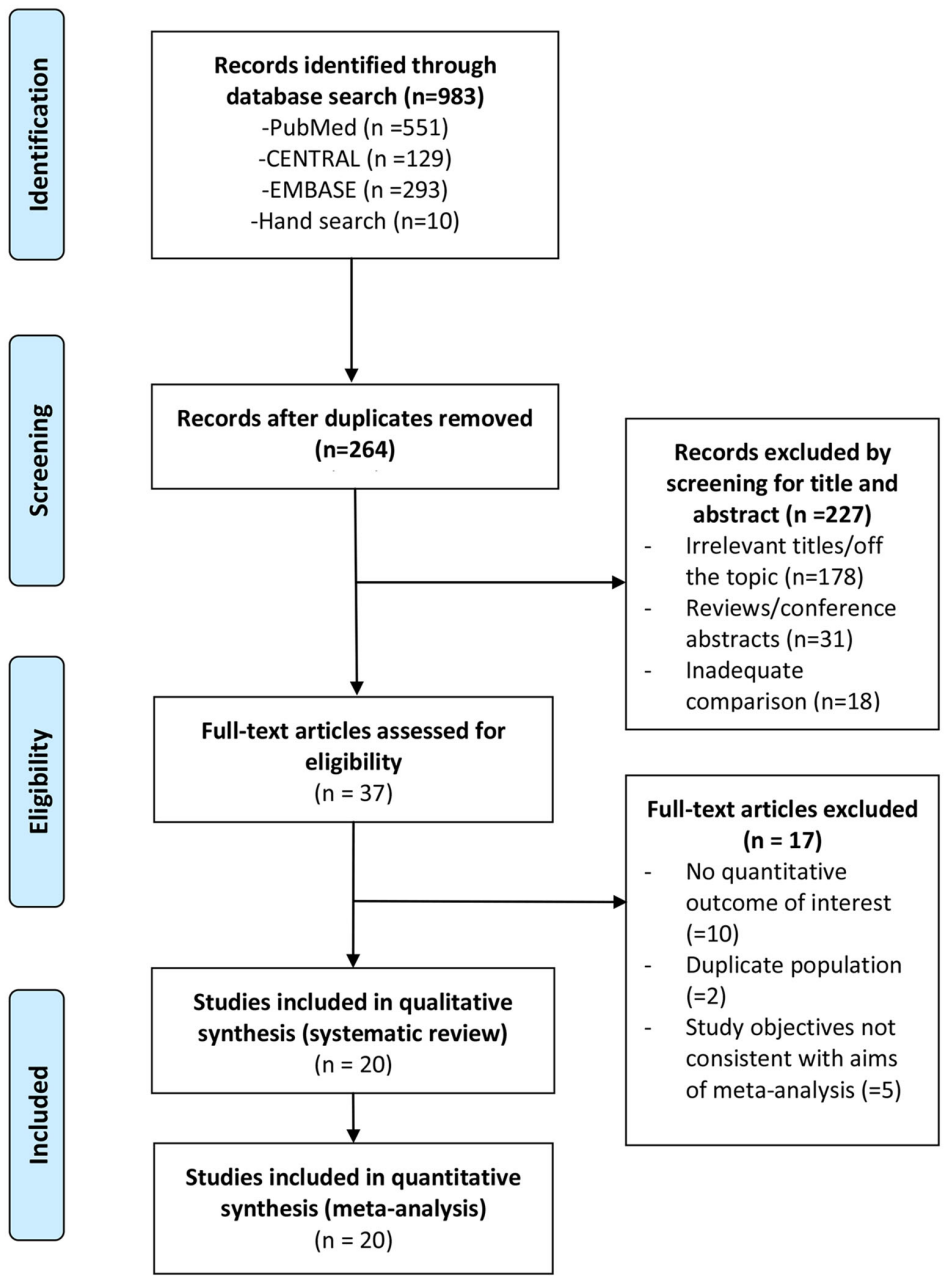
Funnel plots of publication bias assessment for the studies that reported a crude OR of associations between NSAIDs usage and non-union/delayed union.

The Funnel plot for publication bias analysis is shown in **Figure 5**. There was no evidence of publication bias in the included studies according to Egger’s regression test (p = 0.496). Due to the small number of studies that reported an adjusted OR for non-union/delayed union outcome and reoperation, publication bias assessment was not performed (**Figure 5**).

### Sensitivity analyses

Results of the sensitivity analysis performed using the leave-one-out approach are shown in **Supplementary Tables 1**–**3**. The pooled crude OR for non-union/delayed union did not significantly change upon removing any single study, suggesting that none of the studies excessively influenced the pooled estimates. Similarly, the pooled adjusted OR for non-union/delayed union also did not significantly change upon removing any single study.

As for the association between NSAID usage and reoperation, when removing the study by Lindsay et al. (35), the pooled OR became insignificant (OR = 1.42; 95% CI: 0.88–2.28; p = 0.152), indicating that study influenced the meta-analysis result substantially (**Supplementary Tables 1**–**3**).

### Quality assessment

The quality ratings of individual studies as assessed by the NOS are shown in **Table 1**. The overall quality rating indicates that the included studies are of moderate quality.

## Discussion

This updated systematic review and meta-analysis included 20 studies and over 500,000 patients. The meta-analysis investigated the association between NSAIDs and the risk of non-union or delayed union in bone healing, as well as reoperation. The crude analysis, which did not adjust for confounding factors, suggested a significant association of NSAIDs with increased risk in the overall population and certain subgroups, such as adults or patients treated for long bone fractures. However, when the analysis was narrowed to studies that adjusted for confounding variables, the association was no longer statistically significant, indicating that NSAIDs may not significantly impact bone healing. The robustness of this finding was confirmed by sensitivity analysis. Additionally, the initial meta-analysis results showed a significant association between NSAID use and the need for reoperation. However, in the sensitivity analysis, after excluding the largest study from the analysis this association also became statistically insignificant. This indicates that the initial finding might have been influenced by that particular study. Overall, these results suggest that, in real-world settings, NSAIDs might have minimal impact on bone healing. It is important to note, however, that such conclusions should be interpreted with caution and in the context of the relatively small number of studies that reported adjusted ORs, their retrospective design, and the types and dosages of NSAIDs used.

Moderate heterogeneity was detected in most of our pooled analyses, necessitating further discussion. Firstly, although we pooled the adjusted ORs, the analysis includes data from a diverse set of studies with significant variations in demographics data, which might still result in residual confounding. Secondly, variations in the exact fracture sites and comorbid conditions across the included studies might have also contributed to the heterogeneity. Notably, it is known that NSAIDs exert their effects by inhibiting cyclooxygenase-1 (COX-1) and COX-2. Even drugs classified as COX-2 inhibitors have different pharmacokinetics and pharmacodynamics. Accordingly, such variations among the NSAIDs included in this meta-analysis could confound the results and consequently, leading to the statistical insignificance we observed.

The influence of NSAIDs on bone healing represents a nuanced and highly debated issue within the medical research community, which has been the focus of extensive investigation over numerous years. Overall studies have provided inconsistent results. Many other systematic reviews and meta-analysis have attempted to examine the relation between NSAID use and bone healing. In a study published in 2016, Marquez-Lara systematically reviewed literature examining NSAID use and bone healing and categorized the quality of the studies using the modified Coleman Methodology Score (9). Interestingly, studies that demonstrated a negative effect of NSAIDs on bone healing had significantly lower Coleman scores than those that concluded that NSAIDs had no effect on bone healing. In the comprehensive review, a total of two meta-analyses and 22 narrative reviews were examined. Notably, the reviews that concluded that NSAIDs are safe for use referenced a significantly higher number of clinical studies compared with those reviews advocating for the avoidance of NSAIDs. This discrepancy highlights the variance in the breadth of evidence considered in forming conclusions about the safety and impact of NSAIDs on bone healing, suggesting a potential correlation between the volume of supportive clinical data and the perceived safety of NSAIDs in the context of bone healing processes.

In a systematic review conducted by Borgeat et al. in 2018, which scrutinized the literature spanning the preceding 38 years, a total of three prospective Randomized Controlled Trials (RCTs) and 13 retrospective studies that were identified and included in the analysis. A meta-analysis was not be performed because of the marked differences between the studies. The authors concluded that there was not strong evidence that NSAIDs increased the non-union rate; however, the authors also noted that the overall study quality was poor. A prior meta-analysis of the effect of NSAIDs on bone healing rates by Wheatley et al. (13) found that NSAID exposure increased delayed union or non-union (OR = 2.07; 95% CI: 1.19–3.61). Notably, the effect was seen in adults but not in children. Our analysis presents a nuanced perspective that both aligns with and diverges from existing literature. In our analysis, unadjusted pooled results suggested a link between NSAID use and increased risk of bone non-union; on the other hand, using pooled adjusted ORs revealed no association between NSAID use and non-union. These findings underscore the complexity of the relation between NSAID use and bone healing.

A reason for these inconsistent results may be in part due to the complexity of factors influencing outcomes such as the particular drug used and duration of treatment, patient age and the severity of the injury as well as the specific bone injured, and methods used for bone fixation. Some reviews and meta-analysis have focused on the healing of specific bones. Piche et al. (36) performed a systematic review to examine the effect of NSAIDs on spinal fracture healing. Although 1,715 studies were initially screened, only three studies (214 patients) met their inclusion criteria. There was very large heterogeneity among the studies and the results varied markedly: one study reported a 96% healing rate, whereas another reported a 90% non-union rate. Tian et al. (37) studied the factors influencing non-union of tibial fractures. They included 111 studies (41,429 patients) in the analysis and identified 15 factors associated with non-union, and NSAID use was one of the factors associated with non-union. Duchman et al. (38) performed a systematic review and meta-analysis to determine the effect of NSAIDs on tendon-to-bone healing. Three clinical and 10 basic science studies were included in their analysis. One clinical study reported a higher rate of rotator cuff repair failure with a selective NSAID (COX-2) compared with non-selective NSAIDs, whereas analysis of the animal studies found no significant effect of NSAIDs on repair failure.

The current meta-analysis encompassed a diverse cohort, incorporating both pediatric and adult patients. Subsequent subgroup analyses conducted for each demographic revealed a notable finding: among the children population, there was no significant association detected between the use of NSAIDs and the occurrence of bone non-union. This finding aligns with the conclusions drawn in previous literature. Contrary to the results of studies of adults, prior studies of children have overall found that NSAID use has no effect on bone healing. A review of the literature in 2021 by Choo and Nuelle (39) concluded that there is no increased with of non-union in children who are treated with NSAIDs for pain control. A systematic review by Stroud et al. (40) published in 2022 concluded a similar result. The authors included six articles in their final analysis and found that none of the studies reported increased non-union or delayed bone healing in children who received NSAIDs.

We did not conduct separate analyses to explore the association between specific types of NSAIDs and outcomes due to the lack of sufficient data, which precluded the possibility of performing meaningful pooled analyses. Previously, a few of studies have examined the effect of specific NSAIDs on bone healing. For example, Kim et al. (41) reported that short-term use of COX-2 inhibitors had no effect on long-bone fracture healing rates, use for > 3 weeks may be associated with higher rates of non-union or delayed union. In an RCT, Aliuskevicius et al. (42) reported that ibuprofen use had no effect on bone healing of a Colles’ fracture. A recent review of the literature regarding ketorolac and bone healing concluded that there is no evidence short-term use in the peri-operative period has any effect on bone healing, but long-term use may negatively affect fracture healing (43). Given these diverse outcomes, there is a clear need for further research to bridge the gap.

### Strengths and limitations

This comprehensive systematic review and meta-analysis synthesized the evidence concerning the impact of NSAIDs usage on bone healing outcomes and provides a more precise and dependable evaluation of the impact of NSAIDs on bone healing in contrast to prior analyses that might not have fully considered confounding factors. To enhance the reliability of our assessment, we separately pooled both crude and adjusted ORs, which is a major advantage over earlier reviews. In addition, the robustness of the findings is supported by the sensitivity analysis. However, several important limitations should be noted. First, the small number of studies reporting adjusted ORs is a limitation. Additionally, all included studies are of retrospective design, which lowers the evidence level of the meta-analysis compared with those including prospective studies. The included studies encompass a variety of patient conditions, such as degenerative spinal disease and long bone fractures, and patients’ demographic and baseline conditions may vary considerably. Another important issue is that many studies did not specify the type (e.g., COX-1 or COX-2), dosage, and duration of NSAID usage, which might possibly introduce bias into the analytic results. Future meta-analyses or systematic reviews are still warranted once more publications become available to address these shortcomings.

## Conclusion

This updated systematic review and meta-analysis suggests that NSAIDs may have a minimal impact on the risk of non-union or delayed union. However, these findings should be interpreted cautiously due to the relatively limited number of studies included, and the types and dosages of NSAIDs could not be included in the analysis.

## Data Availability

The original contributions presented in the study are included in the article/**Supplementary Material**. Further inquiries can be directed to the corresponding author.
