# Supplementary material for: Do NSAIDs affect bone healing rate, delay union, or cause non-union: an updated systematic review and meta-analysis
Source: Front Endocrinol (Lausanne). 2024 Sep 10;15:1428240. doi: 10.3389/fendo.2024.1428240 (PMC11420001; doi:10.3389/fendo.2024.1428240)

**Supplementary Figure 1.** Subgroup analyses of the associations between NSAIDs usage and non-union or delayed union assessed by crude OR, in adults and in children


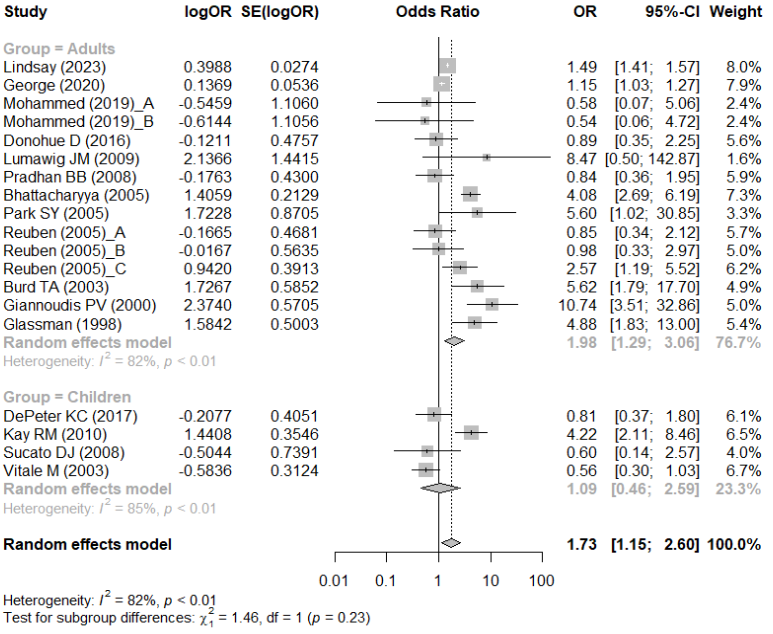


**Supplementary Figure 2.** Subgroup analyses of the associations between NSAID usage and non-union or delayed union assessed by crude OR, according to patient condition.


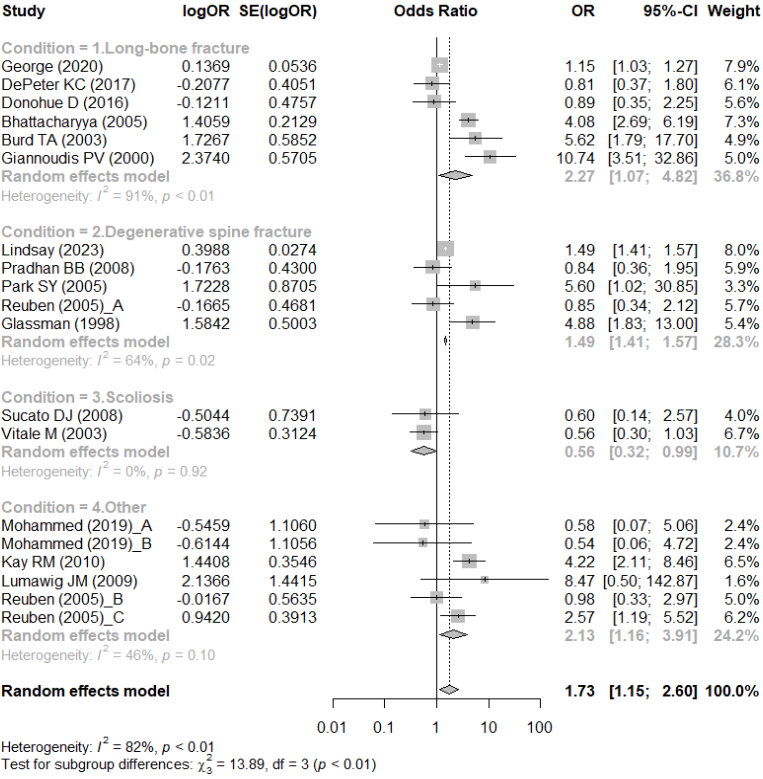

Supplement: Supplementary file 1 [file DataSheet1.docx]
