# Supplementary material for: Do NSAIDs affect bone healing rate, delay union, or cause non-union: an updated systematic review and meta-analysis
Source: Front Endocrinol (Lausanne). 2024 Sep 10;15:1428240. doi: 10.3389/fendo.2024.1428240 (PMC11420001; doi:10.3389/fendo.2024.1428240)
Supplement: Supplementary file 2 [file Table1.docx]

**Supplementary Table 1.** Sensitivity analyses for the association between NSAID usage and non-union/delayed union assessed by crude OR

| Study left out | OR (95% CI) | p-value | tau^2^ | tau | I^2^ |
| --- | --- | --- | --- | --- | --- |
| Lindsay (2023) | 1.76 (1.13-2.73) | 0.012 | 0.604 | 0.777 | 81.8% |
| George (2020) | 1.80 (1.16-2.78) | 0.008 | 0.584 | 0.764 | 78.5% |
| Mohammed (2019)-A | 1.78 (1.18-2.69) | 0.006 | 0.546 | 0.739 | 83.1% |
| Mohammed (2019)-B | 1.78 (1.18-2.70) | 0.006 | 0.544 | 0.737 | 83.0% |
| DePeter (2017) | 1.82 (1.19-2.77) | 0.005 | 0.547 | 0.739 | 82.8% |
| Donohue (2016) | 1.80 (1.18-2.75) | 0.006 | 0.558 | 0.747 | 83.0% |
| Kay (2010) | 1.63 (1.08-2.46) | 0.021 | 0.519 | 0.720 | 81.5% |
| Lumawig (2009) | 1.69 (1.12-2.54) | 0.012 | 0.538 | 0.733 | 82.9% |
| Pradhan (2008) | 1.81 (1.19-2.77) | 0.006 | 0.551 | 0.742 | 82.9% |
| Sucato (2008) | 1.81 (1.20-2.74) | 0.005 | 0.538 | 0.734 | 82.9% |
| Bhattacharyya (2005) | 1.62 (1.07-2.44) | 0.023 | 0.509 | 0.713 | 77.8% |
| Park (2005) | 1.66 (1.10-2.51) | 0.015 | 0.535 | 0.731 | 82.8% |
| Reuben (2005)-A | 1.81 (1.19-2.76) | 0.006 | 0.553 | 0.744 | 83.0% |
| Reuben (2005)-B | 1.79 (1.17-2.73) | 0.007 | 0.565 | 0.752 | 83.1% |
| Reuben (2005)-C | 1.69 (1.10-2.60) | 0.017 | 0.578 | 0.760 | 82.8% |
| Vitale (2003) | 1.88 (1.26-2.80) | 0.002 | 0.469 | 0.685 | 81.5% |
| Burd (2003) | 1.63 (1.08-2.45) | 0.019 | 0.510 | 0.714 | 82.2% |
| Giannoudis (2000) | 1.57 (1.08-2.30) | 0.019 | 0.415 | 0.644 | 80.8% |
| Glassman (1998) | 1.63 (1.08-2.46) | 0.019 | 0.516 | 0.718 | 82.1% |
| Pooled estimate (no study left out) | 1.73 (1.15-2.60) | 0.008 | 0.538 | 0.733 | 82.2% |

OR, odds ratio; CI, confidence interval; NSAIDs, non-steroidal anti-inflammatory drugs.

**Supplementary Table 2.** Sensitivity analyses of pooled adjusted OR of associations between NSAIDs and non-union or delayed union.

| Study left out | OR (95% CI) | p-value | tau^2^ | tau | I^2^ |
| --- | --- | --- | --- | --- | --- |
| George (2020) | 1.49 (0.97-2.29) | 0.066 | 0.043 | 0.208 | 28.5% |
| Jeffcoach (2014) | 1.08 (0.97-1.21) | 0.153 | 0.000 | 0.002 | 0.0% |
| Schemitsch (2012) | 1.11 (0.99-1.24) | 0.063 | 0.000 | 0.000 | 49.8% |
| Reuben (2005)-A | 1.10 (0.99-1.23) | 0.073 | 0.000 | 0.001 | 50.9% |
| Reuben (2006)-B | 1.11 (0.99-1.23) | 0.068 | 0.000 | 0.003 | 50.7% |
| Reuben (2005)-C | 1.10 (0.98-1.22) | 0.102 | 0.000 | 0.000 | 15.8% |
| Pooled estimate (no study left out) | 1.11 (0.99-1.23) | 0.070 | 0.000 | 0.002 | 38.7% |

OR, odds ratio; CI, confidence interval; NSAIDs, non-steroidal anti-inflammatory drugs.

**Supplementary Table 3.** Sensitivity analyses of pooled crude OR of associations between NSAID usage and reoperation.

| Study left out | OR (95% CI) | p-value | tau^2^ | tau | I^2^ |
| --- | --- | --- | --- | --- | --- |
| Lindsay (2023) | 1.42 (0.88-2.28) | 0.152 | 0.000 | 0.002 | 14.20% |
| Blomquist (2014) | 1.98 (1.88-2.07) | < 0.001 | 0.000 | 0.002 | 5.70% |
| Vitale (2003) | 1.98 (1.88-2.07) | < 0.001 | 0.000 | 0.000 | 8.20% |
| Bhandari (2003) | 1.97 (1.88-2.07) | < 0.001 | 0.000 | 0.002 | 51.30% |
| Pooled estimate (no study left out) | 1.97 (1.88-2.07) | < 0.001 | 0.000 | 0.003 | 28.60% |

OR, odds ratio; CI, confidence interval; NSAIDs, non-steroidal anti-inflammatory drugs.
